# Genetic polymorphisms of Wnt/β-catenin pathway genes are associated with the efficacy and toxicities of radiotherapy in patients with nasopharyngeal carcinoma

**DOI:** 10.18632/oncotarget.12754

**Published:** 2016-10-19

**Authors:** Jingjing Yu, Yuling Huang, Lijuan Liu, Jing Wang, Jiye Yin, Lihua Huang, Shaojun Chen, Jingao Li, Hong Yuan, Guoping Yang, Wenyu Liu, Hai Wang, Qi Pei, Chengxian Guo

**Affiliations:** ^1^ Center of Clinical Pharmacology, The Third Xiangya Hospital, Central South University, Changsha, Hunan 410013, China; ^2^ Department of Radiation Oncology, Jiangxi Province Tumor Hospital, Nanchang, Jiangxi 330029, China; ^3^ Department of Pharmacy, Jiangxi Cancer Hospital, Nanchang 330029, China; ^4^ Department of Clinical Pharmacology, Xiangya Hospital, Central South University, Changsha, Hunan 410008, China; ^5^ Center for Medical Experiments, The Third Xiangya Hospital, Central South University, Changsha, Hunan 410013, China; ^6^ Department of Oncology, Fourth Affiliated Hospital, Guangxi Medical University, Liuzhou, Guangxi 545005, China; ^7^ College of Pharmacy, Central South University, Changsha, Hunan 410008, China; ^8^ Xiangya Medical College, Central South University, Changsha, Hunan 410008, China

**Keywords:** nasopharyngeal carcinoma (NPC), Wnt/β-catenin pathway, single nucleotide polymorphism (SNP), curative efficacy, toxic reaction

## Abstract

Radiotherapy (RT) is the normative therapeutic treatment for primary nasopharyngeal carcinoma (NPC). Single nucleotide polymorphisms (SNPs) of genes in Wnt/β-catenin pathway are correlated to the development, prognosis, and treatment benefit of various cancers. However, it has not been established whether SNPs of Wnt/β-catenin pathway are associated with nasopharyngeal tumorigenesis and the efficacy of RT in NPC patients. Therefore, in this study, we aimed to investigate the nine potentially functional SNPs of four genes in the Wnt/β-catenin pathway and genotyped these in 188 NPC patients treated with RT. To achieve this goal, associations between these SNPs and the RT's curative efficacy, as well as acute radiation-induced toxic reaction were determined by multifactorial logistic regression. We observed that catenin beta 1 gene (*CTNNB1*) rs1880481 and rs3864004, and glycogen synthase kinase 3 beta gene (*GSK3β*) rs3755557 polymorphisms were significantly associated with poorer efficacy of RT in NPC patients. Moreover, *GSK3β* rs375557 and adenomatous polyposis coli gene (*APC*) rs454886 polymorphisms were correlated with acute grade 3–4 radiation-induced dermatitis and oral mucositis, respectively. In conclusion, this study suggests that gene polymorphisms of Wnt/β-catenin may be novel prognostic factors for NPC patients treated with RT.

## INTRODUCTION

Nasopharyngeal carcinoma (NPC) is a common malignancy with an obvious ethnic and geographical distributed imbalance in the incidence rate [1]. The annual incidence of NPC in United States is only 0.7 per 100,000, while in southern China it is much greater, with annual rates between 15 and 30 cases per 100,000 in Hong Kong alone [1, 2]. Radiotherapy (RT) is the normative treatment choice for primary NPC patients [2, 3]. Despite advances in RT techniques and development of other efficacious modalities such as surgery and chemotherapy, patients with NPC still have a poor prognosis due to a range of adverse effects and drug resistance [4]. With the development of radiogenomics, a number of studies have demonstrated that dysregulation of gene expression may be useful as independent factors for the curative efficacy as well as the toxicity of RT (with or without concurrent chemotherapy) in NPC patients [5–9]. Moreover, single nucleotide polymorphisms (SNPs) of genes have been validated to be involved in the development, progression and treatment benefit of human cancers [10–12]. For example, one study identified that SNPs of interferon regulatory factor-4 (IRF4) were associated with melanoma development [13]. Another study validated that SNPs of F-box and WD repeat domain containing 7 (FBW7) could be the predictive factor for paclitaxel plus cisplatin chemotherapy in patients with advanced esophageal squamous cell carcinoma [14]. Moreover, SNP of WWOX was found to represent a major predictive factor in gemcitabine-treated pancreatic cancer [15]. Similarly, Li et al. reported that NPC patients with the *XRCC1* 399Arg/Gln genotype were more likely to experience severe acute radiation-induced dermatitis and oral mucositis [16]. Additionally, it has been reported that SNPs of matrix metalloproteinase-1(MMP-1) promoter were associated with risk of radiation-induced lung injury in lung cancer patients with radiotherapy treatment [17].

The Wnt signaling pathway plays a vital role in many fundamental physiological processes [18]. Wnt ligands can direct these processes via a number of signaling pathways. The Wnt/β-catenin pathway, including many components, such as β-catenin, adenomatosis polyposis coli (APC), axin, and glycogen synthase kinase 3 beta (GSK3β), is commonly considered to be the canonical Wnt pathway [19]. The central role of Wnt/β-catenin pathway is β-catenin, which forms a destruction complex with APC, axin, and GSK3β, leading to phosphorylation and subsequent degradation in the absence of Wnt signaling. In the presence of Wnt signaling, β-catenin is uncoupled from the complex and transfers into the cell nucleus, and then activates target genes [20]. Several studies have demonstrated that some mutations, especially SNPs of Wnt/β-catenin pathway genes, are correlated to the occurrence, development and prognosis of various cancers including NPC [21–26]. Jiang et al. found that high expression of Wnt2 was strongly associated with poor clinical outcomes in patients with pancreatic cancer [27]. Furthermore, other studies have found the association between the dysregulation of Wnt/β-catenin pathway and RT in certain cancers [28–30]. For example, Hai et al. explored the impact of transient activation of Wnt/β-catenin signaling on radiation damage to salivary glands in a preclinical model [30]. Their results suggested that concurrent transient activation of the Wnt/β-catenin pathway may reduce the risk of radiation-induced salivary gland dysfunction [30]. However, the relationship between SNPs of Wnt/β-catenin pathway genes and the efficacy of, or the toxic reactions to, RT in NPC patients has not been elucidated.

According to previously published studies [31–38], 9 potentially functional SNPs of 4 key genes in the Wnt/β-catenin pathway, including catenin beta 1(C*TNNB1*, encoding β-catenin) rs4135385, rs1880481, rs3864004; *GSK3*β rs375557; *APC* rs454886, rs3777860; *Wnt2* rs2896218, rs10487362, and rs6947329, were selected in our current study. The purpose of this study was to establish the relationship, if any, between these 9 SNPs of the Wnt/β-catenin pathway genes and the efficacy of RT, as well as acute radiation-induced toxic reaction in NPC patients. We also aimed to find novel genetic markers for predicting NPC patients' response to RT.

## RESULTS

### Clinical characteristics, patients' responses to RT and genotyping

The studied cohort of 188 patients included 130 males and 58 females, with a mean age of 50.7 (ranging from 14 to 81) years old. Serum EBV-DNA of 127 patients (67.6%) were found to be positive. 150 patients (79.8%) were treated with chemotherapy, while the remaining 38 (20.2%) were treated with radiotherapy alone. The general clinical characteristics and outcomes, including curative efficacy and toxic reactions after RT, of the 188 patients with NPC are summarized in Table 1. Although some data were missing, we included 155 patients for the curative efficacy of RT on the lymph node after treatment, 135 patients for the curative efficacy of RT on the primary tumor 3 months after treatment, and 114 patients for the curative efficacy of RT on the lymph node 3 months after treatment. Overall, 19.7% of the patients did not get complete remission (CR) for their primary tumors immediately after RT, while 8.1% failed to get CR for their primary tumors 3 months after RT. Further, 14.2% of the patients did not get CR for their lymph nodes immediately after RT, while 10.5% did not do so 3 months after RT. Regarding toxic reactions, in the 188 patients, 16 (8.5%) experienced grade 3–4 acute radiation-induced dermatitis, 89 (47.3%) presented with grade 3–4 acute radiation-induced oral mucositisand, and 44 (23.4%) suffered from grade 3–4 acute radiation-induced myelosuppression. The results of genotypes of 9 SNPs in the *APC, Wnt2, CTNNB1* and *GSK3*β genes of the 188 patients are shown in Table 2. For one of the 188 patients, we did not obtain the genotype of *Wnt2* rs6947329, and for another patient, we did not obtain the genotypes of *APC* rs3777860 and *CTNNB1* rs3864004. All SNPs were analyzed by Hardy-Weinberg equilibrium. However, the genotype distributions of rs4135385 and rs3777860 polymorphisms were not in accordance with the Hardy-Weinberg equilibrium and excluded in further analysis. All the minor allele frequencies (MAFs) of the residual 7 SNPs were > 10%.

**Table 1:** Summary of the clinical characteristics and outcomes of patients

Characteristics	Patients (%)
Gender	
male	130 (69.1%)
female	58 (30.9%)
Age	
mean ± SD (range)	50.7 ± 11.8 (14–81)
≤ 50	90 (47.9%)
> 50	98 (52.1%)
BMI	
≤ 24.0	123 (65.4%)
> 24.0	65 (34.6)
Family history	
Yes	25 (13.3%)
No	163 (86.7%)
Tumor classification	
T_1_–T_2_	51 (27.1%)
T_3_–T_4_	137 (72.9%)
Lymph node metastasis	
N_0_–N_1_	93 (49.5%)
N_2_–N_3_	95 (50.5%)
Distant metastasis	
M0	182 (96.8%)
M1	6 (3.2%)
Clinical stage	
I–II	26 (13.8%)
III–IV	162 (86.2%)
EBV-DNA	
Positive	127 (67.6%)
Negative	61 (32.4%)
Smoking	
Yes	86 (45.7%)
No	102 (54.3%)
Drinking	
Yes	47 (25.0%)
No	141 (75.0%)
Chemotherapy	
No	38 (20.2%)
Yes	150 (79.8%)
Curative efficacy(Non-CR)	
Primary tumor —after RT	37 (19.7%)
Primary tumor —3 months after RT	11 (8.1%)
lymph node — after RT	22 (14.2%)
lymph node — 3 months after RT	12 (10.5%)
Grade 3–4 radiation- induced toxic reactions	
Dermatitis	16 (8.5%)
Oral mucositis	89 (47.3%)
Myelosuppression	44 (23.4%)

Abbreviations: RT, radiotherapy; BMI, body mass index; CR, complete remission.

**Table 2:** Genotype distribution and MAF of 9 SNPs in *APC, WNT2, CTNNB1* and *GSK3β* genes

Gene	Polymorphic site	Alleles (wild/mutant)	Genotype distribution^a^	HWE	MAF
*WNT2*	rs10487362	G/A	54/91/43	0.695	0.47
*WNT2*	rs2896218	A/G	91/87/10	0.061	0.28
*WNT2*	rs6947329	T/C	55/84/48	0.170	0.48
*APC*	rs3777860	G/A	91/70/26	**0.042**	0.33
*APC*	rs454886	G/A	70/92/26	0.627	0.38
*CTNNB1*	rs4135385	G/A	138/5/45	**< 0.001**	0.25
*CTNNB1*	rsl880481	C/A	107/68/13	0.627	0.25
*CTNNB1*	rs3864004	G/A	101/73/13	0.969	0.26
*GSK3β*	rs3755557	T/A	131/48/9	0.106	0.18

a In the order of wild homozygote / heterozygote / mutant homozygote.

Abbreviations: HWE, Hardy-Weinberg equilibrium; MAF, minor allele frequency.

*P* value < 0.05 is shown in bold.

### Association between clinical factors and responses to RT in NPC patients

All the clinical factors screened out using the chi-square test as covariates in multivariate logistic regression are listed in Supplementary Tables S1 and S2. We found that, compared with the N_0_–N_1_ stage, N_2_–N_3_ stage patients experienced fewer instances of complete remission (CR) for primary tumor (*P* = 0.048) and lymph node (*P* = 0.009), while M_1_ stage patients experienced less incidence of CR for the lymph node (*P* = 0.049) compared with those at M_0_ stage. Patients treated with RT plus chemotherapy had higher risk of grade 3–4 myelosuppresion than these treated with RT alone. We also found that compared with patients younger than 50 years old, those over age 50 showed more positive results at the lymph node both directly after RT (*P* = 0.001) and 3 months after RT (*P* = 0.045).

### Association between selected SNPs of Wnt/β-catenin pathway genes and the efficacy of RT

The relationships between the 7 SNPs and the efficacy of RT on lymph nodes were analyzed by PLINK and all positive results are listed in Table 3. *CTNNB1* rs1880481 polymorphisms were significantly associated with lower efficacy at the lymph node after RT in the recessive model (OR = 4.70, 95% CI = 1.21 − 18.29, *P* = 0.025), while no significant difference was found after adjustment for M stage. After adjustment for age, *CTNNB1* rs1880481 (OR = 5.05, 95% CI = 1.02 – 25.03, *P* = 0.048) and *CTNNB1* rs3864004 (OR = 10.50, 95% CI = 1.03 – 106.60, *P* = 0.047) polymorphisms were strongly related to non-CR at the lymph node 3 months after RT in the recessive model. Patients carrying the minor A allele of *CTNNB1* rs1880481 and rs3864004 showed fewer instances of CR (Supplementary Tables S3 – S6)

**Table 3:** Association between *CTNNB1* rs1880481 and rs3864004 and the efficacy of RT at the lymph node in NPC patients

Time	SNP	Genotype distribution^a^		Model	Unadjusted		Adjusted^b^^,^^c^	
		Non-CR (MAF)	CR (MAF)		OR(95%CI)	*P*	OR(95%CI)	*P*
After RT	rs1880481	4/7/11	6/51/76	allelic	1.67 (0.84–3.30)	0.141	—	—
		(0.34)	(0.24)	addictive	1.67 (0.84–3.33)	0.147	1.71 (0.81–3.60)	0.156
				dominant	1.33 (0.54–3.29)	0.533	1.54 (0.57–4.22)	0.397
				recessive	4.70 (1.21–18.29)	**0.025**	3.79 (0.84–17.17)	0.084
3 months	rs1880481	3/3/6	6/36/60	allelic	1.95 (0.80–4.74)	0.135	—	—
after RT		(0.38)	(0.24)	addictive	1.85 (0.78–4.36)	0.160	1.82 (0.76–4.31)	0.177
				dominant	1.43 (0.43–4.74)	0.560	1.44 (0.43–4.90)	0.555
				recessive	5.33 (1.14–25.01)	**0.034**	5.05 (1.02–25.03)	**0.048**
	rs3864004	2/4/6	3/40/58	allelic	1.70 (0.68–4.21)	0.251	—	—
		(0.33)	(0.23)	addictive	1.78 (0.68–4.68)	0.240	1. 73 (0.62–4.87)	0.299
				dominant	1.35 (0.41–4.47)	0.625	1.19 (0.34–4.18)	0.788
				recessive	6.53 (0.97–43.85)	0.053	10.50 (1.03–106.60)	**0.047**

a In the order of homozygote /heterozygote/ wild type.

b Adjusted for M stage for the association between SNPs and the efficacy of RT at the lymph node.

c Adjusted for age for the association between SNPs and the efficacy of RT at the lymph node 3 months after treatment.

Abbreviations: RT, radiotherapy; CR, complete remission; MAF, minor allele frequency; OR, odds ratio; CI, confidence interval.

*P* value < 0.05 is shown in bold.

### Stratification analysis of association between selected SNPs in Wnt/β-catenin pathway genes and the efficacy of RT

We performed stratification analyses to assess the impacts of these 7 SNPs on the efficacy of RT in NPC patients sorted by age, N stage, and M stage. As shown in Table 4, the A allele of *CTNNB1* rs1880481 and rs3864004 were significantly related with the less-favorable efficacy of RT on the primary tumor 3 months after RT in the allelic, additive and recessive models among patients at the N_0_–N_1_ stage. And *CTNNB1* rs1880481 polymorphism was related to non-CR at the lymph node after RT in the recessive model in patients at the M_0_ (OR = 5.51, 95% CI = 1.40 – 21.77, *P* = 0.015) or the N_2_–N_3_ stage (OR = 4.80, 95% CI = 1.08 – 21.37, *P* = 0.040) stage. In addition, patients at the N_0_–N_1_ stage with A allele of *GSK3*β rs3755557 had less incidence of CR at the lymph node after RT in the allelic (OR = 7.77, 95% CI = 1.42 – 42.58, *P* = 0.007) and additive (OR = 10.65, 95% CI = 1.16 – 97.44, *P* = 0.036) models.

**Table 4:** Stratification analysis of association between SNPs in WNT/β-catenin pathway genes and the efficacy of RT in NPC patients

SNPs	Subgroups (*n*)	Allelic		Addictive		Dominant		Recessive	
		OR (95% CI)	*P*	OR (95% CI)	*P*	OR (95% CI)	*P*	OR (95% CI)	*P*
At the primary tumor 3 months after treatment									
rs1880481	N0–N1 (63)	12.91 (1.28–129.90)	**0.006**	27.06 (1.32–554.60)	**0.032**	NA	0.996	60.00 (1.99–1805.00)	**0.018**
rs3864004	N0–N1 (62)	10.33 (1.03–103.40)	**0.016**	29.75 (1.31–675.60)	**0.033**	NA	0.996	59.00 (1.96–1776.00)	**0.019**
At the lymph node after treatment									
rs1880481	M0 (149)	1.82 (0.89–3.74)	0.097	1.83 (0.88–3.80)	0.103	1.42 (0.54–3.74)	0.474	5.51 (1.40–21.77)	**0.015**
	N2–N3 (95)	1.44 (0.66–3.12)	0.362	1.39 (0.66–2.94)	0.386	0.95 (0.34–2.62)	0.918	4.80 (1.08–21.37)	**0.040**
rs3755557	N0–N1 (60)	7.77 (1.42–42.58)	**0.007**	10.65 (1.16–97.44)	**0.036**	6.77 (0.57–80.70)	0.131	NA	0.999

Abbreviations: OR, odds ratio; CI, confidence interval; NA, not applicable. *P* value < 0.05 is shown in bold.

### Association between selected SNPs of the Wnt/β-catenin pathway genes and acute grade 3–4 radiation-induced toxic reactions

The associations between selected 7 SNPs in Wnt/β-catenin pathway genes and acute grade 3–4 radiation-induced toxic reactions were calculated by PLINK (Table 5). The *GSK3*β rs3755557 polymorphism was significantly correlated with the acute grade 3–4 radiation-induced dermatitis in the allelic model (OR = 2.34, 95% CI = 1.05 – 5.21, *P* = 0.033). Patients with the minor A allele of rs3755557 were less resistant to acute grade 3–4 radiation-induced dermatitis. The *APC* rs454886 polymorphism was significantly associated with acute grade 3–4 radiation-induced oral mucositis in additive (OR = 1.57, 95% CI = 1.01 – 2.43, *P* = 0.045) and recessive models (OR = 2.54, 95% CI = 1.06 – 6.13, *P* = 0.038) after adjustment for BMI. Individuals carrying the minor A allele of the rs454886 polymorphism had an increased risk of acute grade 3–4 radiation-induced oral mucositis. None of these 7 SNPs in the Wnt/β-catenin pathway genes was found to have significant association with acute radiation-induced myelosuppression (Supplementary Table S9). And none of the 7 SNPs was found significantly associated with 3 kinds of acute radiation-induced toxic reaction in stratification analyses.

**Table 5:** Association between *GSK3β* rs3755557 and *APC* rs454886 and the acute grade 3–4 radiation-induced toxic reactions

Radiation-induced toxic reactions	SNP	Genotype distribution^a^		Model	unadjusted		Adjusted^b^	
		Grade 3–4 (MAF)	Grade 0–2 (MAF)		OR (95% CI)	*P*	OR (95% CI)	*P*
Grade 3–4 dermatitis	rs3755557	2/6/8	7/42/123	allelic	2.34 (1.05–5.21)	**0.033**	—	—
		(0.31)	(0.16)	addictive	2.13 (1.00–4.55)	0.051	—	—
				dominant	2.51 (0.89 –7.06)	0.081	—	—
				recessive	3.37 (0.64–17.77)	0.153	—	—
Grade 3–4 oral mucositis	rs454886	17/43/29	9/49/41	allelic	1.49 ((0.98–2.26)	0.061	—	—
		(0.43)	(0.34)	addictive	1.52 (0.99–2.34)	0.058	1.57 (1.01–2.43)	**0.045**
				dominant	1.46 (0.81–2.66)	0.212	1.50 (0.82–2.73)	0.191
				recessive	2.36 (0.99–5.61)	0.052	2.54 (1.06–6.13)	**0.038**

a In the order of wild homozygote /heterozygote/ wild type.

b Adjusted for BMI.

Abbreviations: MAF, minor allele frequency; OR, odds ratio; CI, confidence interval; *P* value < 0.05 is shown in bold.

## DISCUSSION

Emerging evidence has revealed that β-catenin plays an oncogenic role in the regulations of proliferation, differentiation, development and tissue regeneration, and also functions as a transcriptional co-activator of the Wnt/β-catenin pathway [39–42]. For instance, the tumor growth of NPC was markedly attenuated when the expression of *CTNNB1* was suppressed, which indicated that β-catenin may play an important oncogenic role in the migration and proliferation of NPC cells [33]. Importantly, genetic variants in the Wnt/β-catenin pathway could be predictors of cancer risk in multiple human malignancies [43–48]. In this study, we examined the association of 7 SNPs in the Wnt/β-catenin pathway genes and the efficacy and radiation-induced toxic reactions in 188 NPC patients treated with RT (with or without chemotherapy). Our results showed that 3 SNPs (*CTNNB1* rs1880481 and rs3864004, and *GSK3*β rs375557) were correlated to the efficacy of RT, and 2 SNPs (*APC* rs454886 and *GSK3*β rs375557) were correlated with acute grade 3–4 radiation-induced toxic reactions in NPC patients.

In the present study, the results of logistic regression indicated that patients with the minor A allele of *CTNNB1* rs1880481 and rs3864004 polymorphisms obtained less favorable outcomes with RT in NPC. Since the rs1880481 was identified in the intron region of *CTNNB1*, it may up-regulate the expression β-catenin by affecting the mRNA splicing, localization and stability [49]. Another conceivable mechanism is that the real functional variant mediating this process is not the SNP rs1880481 but another variant in linkage disequilibrium (LD) with it [50]. To our knowledge, there has no report about the correlation of rs1880481 polymorphism and development and prognosis of NPC. Without a doubt, more studies about the rs1880481 and potential functional polymorphism sites in LD are requisite to verify the exact mechanism. The rs3864004 was located in the promoter region of the *CTNNB1* and its polymorphism has been reported to be associated with development and survival of hepatocellular carcinoma [51]. Consistent with this, we found that *CTNNB1* rs3864004 was significantly related with poorer efficacy at lymph node after RT in NPC patients (AA *vs* AG+GG: aOR = 10.50, *P* = 0.047). Moreover, our results showed that *CTNNB1* rs3864004 was also associated with the efficacy at primary tumor in NPC patients at N_0_–N_1_ stage. Along with previous study [31], our findings indicated that rs3864004 polymorphism may stimulate β-catenin expression and lead to the accumulation of β-catenin, which could subsequently promote the proliferation of NPC cells and weakens the efficacy of RT.

GSK3β is a crucial regulatory kinase interacting with multiple pathways in addition to the Wnt/β-catenin pathway, which controls various physiological processes such as neurodevelopment and cellular differentiation, proliferation, survival, and apoptosis [52, 53]. The results of subgroup analyses showed that *GSK3*β rs3755557 was associated with reduced efficacy of RT at the lymph node in the allelic (OR = 7.77, *P* = 0.007) and additive (OR = 10.65, *P* = 0.036) models in the N_0_–N_1_ stage patients. Moreover, the A allele of *GSK3*β rs3755557 was also correlated to increased risk of grade 3–4 radiation-induced dermatitis compared with T allele (OR = 2.34, *P* = 0.033) in our study. These results suggested that the polymorphism of *GSK3*β rs3755557 may inhibit the expression of GSK3β, leading to the aggregation of β-catenin, the activation of Wnt/β-catenin signaling, and higher resistance to RT. Our study is the first time to show a relationship between *GSK3*β rs3755557 and radiation-induced dermatitis, but further investigation is needed to delineate the precise mechanism. As a tumor suppressor, APC protects β-catenin from dephosphorylation and enhances β-catenin degradation. Wang et al. reported that the rs454886 polymorphism in *APC* was strongly correlated to an increased risk of breast cancer [54]. We also found that *APC* rs454886 was associated with acute grade 3–4 radiation-induced oral mucositis in the additive (OR = 1.57, *P* = 0.045) and recessive (OR = 2.54, *P* = 0.038) models after adjustment. Further studies are required to elucidate this association between *APC* and radiation-induced toxic reaction in NPC.

The advantage of our study lies in the fact that it is the first time to explore the impact of SNPs in genes of the Wnt/β-catenin pathway on the curative efficacy of RT, as well as on radiation-induced toxic reactions in NPC patients. However, one limitation of this study was its sample size, particularly in relation to our stratification analysis. Therefore, replication studies are required to validate our results in more independent subjects. In conclusion, we found that *CTNNB1* rs1880481, *CTNNB1* rs3864004, and *GSK3B* rs375557 were correlative with the curative efficacy of RT, and that *GSK3*β rs375557 and *APC* rs454886 were correlative with acute grade 3–4 radiation-induced dermatitis and oral mucositis. These SNPs may be potential prognostic markers for NPC patients treated with RT.

## MATERIALS AND METHODS

### Patient selection

The present study's protocol was approved by the ethics committee of the Cancer Hospital of Jiangxi Province (China) and registered online through the website of the Chinese Clinical Trial Registry (www.chictr.org.cn, registration number: ChiCTR-OPC-14005257). All eligible patients signed informed consent before participating in this study. The study initially approached 236 NPC patients treated with RT in the Cancer Hospital of Jiangxi Province between April 2014 and September 2015. Patients were enrolled if they met the following criteria: a diagnosis of NPC; performance status score ≦ 1; no severe disorders of heart, lung, liver or kidney; and no prior treatment with other anticancer therapies before undergoing radiotherapy or chemotherapy, ≧1 measurable lesions. The exclusion criteria were: active infection, pregnancy or lactation, previous or concomitant malignancies and absence of any follow-up. We recorded the demographics and clinical data of enrolled patients including age, gender, and smoking and drinking habits, TNM (7th UICC) stage and comorbidities. Ultimately, 188 patients were included in the study and its subsequent statistical analysis.

### Efficacy regimen

A total of 38 patients were treated with RT alone and the remaining 150 were treated with RT and chemotherapy. All patients were treated with intensity-modulated radiation-therapy (IMRT) technique. The prescribed dosage of radiation therapy was 66 – 70 Gy in 30 – 33 fractions for nasopharyngeal primary focus and the cervical positive lymph nodes and 54 – 60 Gy in 30 – 33 fractions for cervical drainage region, respectively. One fraction was performed daily and five fractions weekly. Induction chemotherapy and concurrent chemotherapy were all performed with platinum-based chemotherapy regimens.

### Evaluations of efficacy and toxic reactions

All patients underwent magnetic resonance imaging (MRI) directly after treatment and 3 months later after treatment. Treatment efficacies at the primary tumor and lymph node were evaluated in line with the response evaluation criteria in solid tumors (RECIST) by the World Health Organization (WHO). RECIST defines treatment efficacy, based on tumor volume, as: complete remission (CR), partial remission (PR), stable disease (SD), and progression disease (PD). In this study we subdivided the subjects into two groups: CR and non-CR (consisting of PR, SD and PD).

Acute radiation-induced toxic reactions including dermatitis, oral mucositis and myelosuppression were recorded and evaluated according to the acute radiation toxicity grading criteria of the Radiation Therapy Oncology Group or European Organization for Research and Efficacy of Cancer (RTOG/EORTC). For each toxic reaction, patients were subdivided into a ‘non-sensitive or mildly radiosensitive’ group (grade 0–2) and a ‘highly radiosensitive’ group (grade 3–4). All acute toxic reactions were observed once a day from the first day to the end of RT.

### DNA extraction and genotyping assays

Peripheral blood samples of 3 mL were taken before radiotherapy and stored in EDTA anti-coagulative tubes at −20°C. Genetic DNA of all patients was extracted using Wizard Genomic DNA Purification Kit (Promega, Madison, USA). The genotyping for 9 SNPs of the Wnt/β-catenin pathway genes was conducted by Sequenom MassARRAY system (Sequenom, San Diego, California, USA). Primers were designed by using Assay Designer 3.1 and the details about primer sequence for each SNP site were listed in Supplementary Table S10. The polymerase chain reaction (PCR) scheme was defined by an initial denaturation at 94°C for 15 min, followed by 45 cycles for 20 s at 94°C, 30 s at 56°C, 1 min at 72°C, with a final elongation at 72°C for 3 min. The products of PCR were purified in shrimp alkaline phosphatase (SAP), which was performed by incubating at 37°C for 40 min, followed by 85°C for 5 min. A extension reaction was performed using extension primers and the extension products were desalted by the addition of a cation exchange resin. The desalination products were centrifuged at 4000r for 4 min before transferred in CHIP. Data from CHIP was analyzed by Typer Analyzer.

### Statistical analysis

Deviations from Hardy-Weinberg equilibrium were calculated using χ2 analysis. Univariate logistic regression was performed to determine the association of each SNP of *APC*, *WNT2*, *CTNNB1*, and *GSK3B* with the curative efficacy at the primary tumor and lymph node. Likewise, the association between these 9 SNPs and the indicators for high radiosensitivity such as dermatitis, oral mucositis, and myelosuppression grade 3–4 was calculated by univariate logistic regression. The chi-square test was used to determine the associations between the two alleles of each SNP and the efficacy and toxic reaction.

Continuous variables such as age and BMI were switched to binary variables. The effects of clinical factors such as sex, age, BMI, TNM cancer stage (UJCC), smoking and drinking habits, family history, and receipt or lack of chemotherapy on efficacy and toxic reactions were computed using chi-square test, separately. Only those factors with a *P* value < 0.1 in the chi-square test were considered to be adjusted factors warranted further multivariate analysis. Subsequently, multivariate logistic regression was carried out to evaluate the effect of each SNP on non-CR at the primary tumor and lymph node as well as grade 3–4 dermatitis, oral mucositis, and myelosuppression, by computing the odds ratio (OR) and the corresponding 95% confidence interval (CI), with CR at the primary tumor and lymph node as well as grade 0–2 dermatitis, oral mucositis, and myelosuppression as the reference, respectively. In addition, stratification analyses were performed to characterize the associations between SNPs and RT efficacy and toxic reactions in some subgroups when the corresponding clinical factor had *P* value < 0.1 in the chi-square test. The difference was considered statistically significant when the *P* value < 0.05. All aforementioned statistical analyses were performed by SPSS18.0 (IBM, Armonk, New York, USA) and PLINK 1.07 (http://pngu.mgh.harvard.edu/~purcell/plink/).

## SUPPLEMENTARY MATERIALS


