# Supplementary material for: Genetic polymorphisms of Wnt/β-catenin pathway genes are associated with the efficacy and toxicities of radiotherapy in patients with nasopharyngeal carcinoma
Source: Oncotarget. 2016 Oct 19;7(50):82528–37. doi: 10.18632/oncotarget.12754 (PMC5347711; doi:10.18632/oncotarget.12754)
Supplement: Supplementary file 1 [file oncotarget-07-82528-s001.pdf]

Genetic polymorphisms of Wnt/β-catenin pathway genes are associated with the efficacy and toxicities of radiotherapy in patients with nasopharyngeal carcinoma

Supplementary Materials

Supplementary Table S1: Association between clinical factors and the efficacy of RT directly after treatment and 3 months after treatment in patients with NPC

Clinical Characteristics	After treatment						3 months after treatment					
	primary tumor (*n* = 188)			lymph node (*n* = 155)			primary tumor (*n* = 135)			lymph node (*n* = 114)		
	CR	Non-CR	*P*	CR	Non-CR	*P*	CR	Non-CR	*P*	CR	Non-CR	*P*
Age												
≤ 50	72	18	0.916	60	18	**0.001**	62	7	0.386	54	10	**0.045**
> 50	79	19		73	4		62	4		48	2	
Gender												
male	107	23	0.305	93	14	0.555	86	8	1.000	70	8	1.000
female	44	14		40	8		38	3		32	4	
Drinking												
Yes	42	5	**0.072**	34	4	0.456	31	3	1.000	26	0	0.104
No	109	32		99	18		93	8		76	12	
Smoking												
Yes	73	13	0.148	59	11	0.622	56	6	0.549	46	6	0.747
No	78	24		74	11		68	5		56	6	
Family history												
Yes	20	5	1.000	18	1	0.401	21	1	0.803	17	0	0.269
No	131	32		115	21		103	10		85	12	
BMI												
≤ 24.0	101	22	0.395	90	12	0.229	80	7	1.000	66	7	0.907
> 24.0	50	15		43	10		44	4		36	5	
Chemotherapy												
No	32	6	0.499	21	4	1.000	24	1	0.664	13	2	1.000
Yes	119	31		112	18		100	10		89	10	
Tumor classification												
T_1–T_2	45	6	**0.096**	32	7	0.437	35	1	0.308	23	3	1.000
T_3–T_4	106	31		101	15		89	10		79	9	
Lymph node metastasis												
N_0–N_1	73	20	0.534	57	3	**0.009**	61	2	**0.048**	39	3	0.560
N_2–N_3	78	17		76	19		63	9		63	9	
Distant metastasis												
M_0	147	35	0.739	130	19	**0.049**	120	10	0.351	98	11	0.433
M_1	4	2		3	3		4	1		4	1	
Clinical stage												
I–II	21	5	0.950	12	2	1.000	19	1	0.909	9	1	1.000
III–IV	130	32		121	20		105	10		93	11	
EBV-DNA												
Negative	46	15	0.241	35	4	0.415	43	1	0.162	26	3	1.000
Positive	105	22		98	18		81	10		76	9	

Abbreviations: RT, radiotherapy; BMI, body mass index. *P* value < 0.1 is shown in bold.

Supplementary Table S2: The association between clinical factors and the acute radiation-induced toxic reactions

Clinical characteristics	Dermatitis (*n* = 188)			Oral mucositis (*n* = 188)			Myelosuppression (*n* = 188)		
	Grade 0–2	Grade 3–4	*P*	Grade 0–2	Grade 3–4	*P*	Grade 0–2	Grade 3–4	*P*
Age									
≤ 50	81	9	0.483	49	41	0.639	67	23	0.504
> 50	91	7		50	48		77	21	
Gender									
male	119	11		66	64	0.437	102	28	0.366
female	53	5	0.971	33	25		42	16	
Drinking									
Yes	43	4	1.000	23	24	0.555	36	11	1.000
No	129	12		76	65		108	33	
Smoking			0.378						
Yes	77	9		44	42	0.706	69	17	0.280
No	95	7		55	47		75	27	
Family history									
Yes	23	2	0.922	14	11	0.719	19	6	0.940
No	149	14		85	78		125	38	
BMI									
≤ 24.0	115	8	0.175	59	64	**0.076**	91	32	0.245
> 24.0	57	8		40	25		53	12	
Chemotherapy									
No	35	3	0.879	21	17	0.719	37	1	**0.001**
Yes	137	13		78	72		107	43	
Tumor classification									
T_1–T_2	46	5	0.698	31	20	0.173	44	7	**0.056**
T_3–T_4	126	11		68	69		100	37	
Lymph node metastasis									
N_0–N_1	86	7	0.632	49	44	0.994	71	22	0.936
N_2–N_3	86	9		50	45		73	22	
Distant metastasis									
M_0	167	15	0.467	96	86	1.000	139	43	0.692
M_1	5	1		3	3		5	1	
Clinical stage									
I–II	23	3	0.551	14	12	0.896	22	4	0.298
III–IV	149	13		85	77		122	40	
EBV–DNA									
Negative	57	4	0.506	31	30	0.726	47	14	0.919
Positive	115	12		68	59		97	30	

Abbreviations: RT, radiotherapy; BMI, body mass index. *P* value < 0.1 is shown in bold.

Supplementary Table S3: Association between SNPs in WNT/β-catenin pathway genes and the efficacy of RT at the primary tumor after treatment

SNP	Genotype distribution[a]		Model	Unadjusted		Adjusted[b]	
	non-CR (MAF)	CR (MAF)		OR (95% CI)	*P*	OR (95% CI)	*P*
rs10487362	6/23/8 (0.47)	37/68/46 (0.47)	allelic	1.01 (0.61–1.68)	0.966	—	—
			additive	1.01 (0.61–1.67)	0.966	0.99 (0.59–1.67)	0.968
			dominant	1.59 (0.67–3.74)	0.290	1.43 (0.60–3.43)	0.417
			recessive	0.60 (0.23–1.54)	0.286	0.63 (0.24–1.66)	0.350
rs2896218	2/19/16 (0.31)	8/68/75 (0.28)	allelic	1.17 (0.67–2.03)	0.577	—	—
			additive	1.20 (0.66–2.19)	0.549	1.22 (0.66–2.26)	0.530
			dominant	1.30 (0.63–2.67)	0.484	1.28 (0.61–2.68)	0.509
			recessive	1.02 (0.21–5.02)	0.979	1.16 (0.22–5.98)	0.861
rs6947329	9/16/11 (0.47)	38/69/44 (0.48)	allelic	0.97 (0.58–1.62)	0.904	—	—
			additive	0.97 (0.59–1.59)	0.908	0.99 (0.59–1.64)	0.961
			dominant	0.93 (0.42–2.06)	0.867	0.91 (0.41–2.03)	0.814
			recessive	0.99 (0.43–2.29)	0.984	1.08 (0.46–2.53)	0.867
rs454886	4/21/12 (0.39)	31/3/117 (0.38)	allelic	1.05 (0.62–1.77)	0.860	—	—
			additive	1.05 (0.62–1.79)	0.858	1.01 (0.59–1.73)	0.976
			dominant	1.30 (0.61–2.79)	0.501	1.26 (0.58–2.73)	0.562
			recessive	0.71 (0.23–2.20)	0.554	0.65 (0.21–2.05)	0.463
rs1880481	3/18/16 (0.32)	9/51/91 (0.23)	allelic	1.62 (0.93–2.83)	0.087	—	—
			additive	1.62 (0.92–2.83)	0.092	1.61 (0.91–2.87)	0.104
			dominant	1.99 (0.96–4.12)	0.064	2.05 (0.97–4.32)	0.060
			recessive	1.39 (0.36–5.42)	0.633	1.25 (0.31–5.04)	0.753
rs3864004	3/15/19 (0.28)	4/63/83 (0.24)	allelic	1.28 (0.72–2.26)	0.399	—	—
			additive	1.33 (0.72–2.46)	0.369	1.3 (0.7–2.42)	0.414
			dominant	1.17 (0.57–2.41)	0.663	1.18 (0.56–2.45)	0.665
			recessive	3.22 (0.69–15.06)	0.137	2.74 (0.56–13.4)	0.214
rs3755557	1/9/27 (0.15)	8/39/104 (0.18)	allelic	0.78 (0.39–1.59)	0.498	—	—
			additive	0.80 (0.41–1.57)	0.522	0.84 (0.43–1.67)	0.627
			dominant	0.82 (0.37–1.83)	0.627	0.87 (0.38–1.97)	0.732
			recessive	0.50 (0.06–4.10)	0.516	0.55 (0.06–4.62)	0.578

[a]In the order of homozygote / heterozygote / wild type.
[b]Adjusted for T stage and drinking status.
Abbreviations: MAF, minor allele frequency; OR, odds ratio; CI, confidence interval. *P* value < 0.05 is shown in bold.

Supplementary Table S4: Association between SNPs in WNT/β-catenin pathway genes and the efficacy of RT at the primary tumor 3 months after treatment

SNP	Genotype distribution[a]		Model	Unadjusted		Adjusted[b]	
	non-CR(MAF)	CR (MAF)		OR (95% CI)	*P*	OR (95% CI)	*P*
rs10487362	3/4/4 (0.45)	30/63/31 (0.50)	allelic	0.85 (0.35–2.03)	0.710	—	—
			additive	0.85 (0.35–2.03)	0.711	0.85 (0.35–2.03)	0.711
			dominant	0.58 (0.16–2.13)	0.414	0.58 (0.16–2.13)	0.414
			recessive	1.18 (0.29–4.71)	0.820	1.18 (0.29–4.71)	0.820
rs2896218	1/3/7 (0.23)	4/60/60 (0.27)	allelic	0.78 (0.28–2.19)	0.635	—	—
			additive	0.74 (0.24–2.29)	0.600	0.74 (0.24–2.29)	0.600
			dominant	0.54 (0.15–1.92)	0.338	0.54 (0.15–1.92)	0.338
			recessive	3.00 (0.31–29.46)	0.346	3.00 (0.31–29.46)	0.346
rs6947329	3/2/6 (0.36)	33/57/33 (0.50)	allelic	0.57 (0.23–1.41)	0.220	—	—
			additive	0.60 (0.26–1.43)	0.253	0.6 (0.26–1.43)	0.253
			dominant	0.31 (0.09–1.07)	0.063	0.31 (0.09–1.07)	0.063
			recessive	1.02 (0.26–4.09)	0.975	1.02 (0.26–4.09)	0.975
rs454886	0/6/5 (0.27)	20/61/43 (0.41)	allelic	0.55 (0.21–1.44)	0.216	—	—
			additive	0.53 (0.20–1.44)	0.214	0.53 (0.20–1.44)	0.214
			dominant	0.64 (0.18–2.21)	0.477	0.64 (0.18–2.21)	0.477
			recessive	—	0.997	—	0.997
rs1880481	2/4/5 (0.36)	7/43/74 (0.23)	allelic	1.92 (0.76–4.79)	0.160	—	—
			additive	1.86 (0.75–4.59)	0.177	1.86 (0.75–4.59)	0.177
			dominant	1.78 (0.51–6.14)	0.364	1.78 (0.51–6.14)	0.364
			recessive	3.71 (0.67–20.57)	0.133	3.71 (0.67–20.57)	0.133
rs3864004	1/4/6 (0.27)	4/49/70 (0.23)	allelic	1.24 (0.46–3.33)	0.664	—	—
			additive	1.28 (0.45–3.63)	0.648	1.28 (0.45–3.63)	0.648
			dominant	1.10 (0.32–3.80)	0.880	1.10 (0.32–3.80)	0.880
			recessive	2.98 (0.30–29.21)	0.350	2.98 (0.3–29.21)	0.350
rs3755557	1/2/8 (0.18)	7/34/83 (0.19)	allelic	0.93 (0.30–2.86)	0.894	—	—
			additive	0.93 (0.32–2.69)	0.901	0.93 (0.32–2.69)	0.901
			dominant	0.76 (0.19–3.01)	0.695	0.76 (0.19–3.01)	0.695
			recessive	1.67 (0.19–14.97)	0.646	1.67 (0.19–14.97)	0.646

[a]In the order of homozygote / heterozygote / wild type; [b] Adjusted for N stage.
Abbreviations: MAF, minor allele frequency; OR, odds ratio; CI, confidence interval. *P* value < 0.05 is shown in bold.

Supplementary Table S5: Association between SNPs in WNT/β-catenin pathway genes with the efficacy of RT at the lymph node after treatment

SNP	Genotype distribution[a]		Model	Unadjusted		Adjusted[b]	
	non-CR (MAF)	CR (MAF)		OR (95% CI)	*P*	OR (95% CI)	*P*
rs10487362	7/9/6 (0.52)	27/64/42 (0.44)	allelic	1.37 (0.73–2.60)	0.329	—	—
			additive	1.35 (0.72–2.53)	0.343	1.51 (0.76–2.99)	0.235
			dominant	1.23 (0.45–3.37)	0.686	1.44 (0.48–4.35)	0.518
			recessive	1.83 (0.68–4.94)	0.231	2.14 (0.71–6.44)	0.175
rs2896218	2/8/12 (0.27)	7/63/63 (0.29)	allelic	0.92 (0.45–1.88)	0.820	—	—
			additive	0.91 (0.43–1.95)	0.809	0.88 (0.38–2.02)	0.760
			dominant	0.75 (0.30–1.86)	0.534	0.68 (0.25–1.83)	0.441
			recessive	1.80 (0.35–9.29)	0.483	2.22 (0.36–13.82)	0.391
rs6947329	6/10/6 (0.50)	33/60/39 (0.48)	allelic	1.1 (0.58–2.07)	0.780	—	—
			additive	1.09 (0.59–2.00)	0.789	1.15 (0.60–2.21)	0.681
			dominant	1.12 (0.41–3.07)	0.828	1.34 (0.44–4.11)	0.606
			recessive	1.13 (0.41–3.11)	0.821	1.10 (0.37–3.30)	0.868
rs454886	3/11/8 (0.39)	17/63/53 (0.36)	allelic	1.10 (0.57–2.11)	0.782	—	—
			additive	1.10 (0.57–2.14)	0.779	1.42 (0.67–3.00)	0.365
			dominant	1.16 (0.45–2.96)	0.757	1.62 (0.56–4.66)	0.372
			recessive	1.08 (0.29–4.03)	0.912	1.45 (0.34–6.18)	0.619
rs1880481	4/7/11 (0.34)	6/51/76 (0.24)	allelic	1.67 (0.84–3.30)	0.141	—	—
			additive	1.67 (0.84–3.33)	0.147	1.71 (0.81–3.6)	0.156
			dominant	1.33 (0.54–3.29)	0.533	1.54 (0.57–4.22)	0.397
			recessive	4.70 (1.21–18.29)	0.025	3.79 (0.84–17.17)	0.084
rs3864004	1/9/12 (0.25)	5/55/72 (0.25)	allelic	1.02 (0.49–2.13)	0.957	—	—
			additive	1.02 (0.47–2.25)	0.954	1.16 (0.49–2.76)	0.735
			dominant	1.00 (0.40–2.48)	1.000	1.16 (0.42–3.17)	0.775
			recessive	1.21 (0.13–10.87)	0.865	1.38 (0.13–14.84)	0.792
rs3755557	1/6/15 (0.18)	6/31/96 (0.16)	allelic	1.15 (0.50–2.65)	0.738	—	—
			additive	1.13 (0.52–2.47)	0.754	1.11 (0.47–2.63)	0.815
			dominant	1.21 (0.46–3.21)	0.700	1.13 (0.39–3.28)	0.824
			recessive	1.01 (0.12–8.80)	0.994	1.18 (0.12–11.97)	0.889

[a]In the order of homozygote / heterozygote / wild type.

[b]Adjusted for N stage, M stage and age.

Abbreviations: MAF, minor allele frequency; OR, odds ratio; CI, confidence interval. P value < 0.05 is shown in bold.

Supplementary Table S6: Association between SNPs in WNT/β-catenin pathway genes and the efficacy of RT at the lymph node 3 months after treatment

SNP	Genotype distribution[a]		Model	Unadjusted		Adjusted[b]	
	non-CR (MAF)	CR (MAF)		OR (95% CI)	*P*	OR (95% CI)	*P*
rs10487362	4/5/3 (0.54)	24/50/28 (0.48)	allelic	1.28 (0.55–2.99)	0.570	—	—
			additive	1.27 (0.55–2.93)	0.577	1.38 (0.59–3.26)	0.462
			dominant	1.14 (0.29–4.50)	0.857	1.25 (0.31–5.06)	0.758
			recessive	1.63 (0.45–5.87)	0.459	1.86 (0.49–6.97)	0.360
rs2896218	0/5/7 (0.21)	4/48/50 (0.27)	allelic	0.70 (0.25–1.95)	0.489	—	—
			additive	0.65 (0.21–1.98)	0.445	0.65 (0.21–2.00)	0.456
			dominant	0.69 (0.2–2.31)	0.543	0.71 (0.21–2.43)	0.584
			recessive	—	0.999	NA	0.999
rs6947329	4/4/4 (0.50)	28/45/28 (0.50)	allelic	1.00 (0.43–2.33)	1.000	—	—
			additive	1.00 (0.45–2.22)	1.000	0.98 (0.44–2.19)	0.962
			dominant	0.77 (0.21–2.75)	0.684	0.75 (0.21–2.77)	0.671
			recessive	1.30 (0.36–4.67)	0.684	1.26 (0.34–4.61)	0.732
rs454886	2/5/5 (0.38)	15/50/37 (0.39)	allelic	0.93 (0.39–2.23)	0.871	—	—
			additive	0.93 (0.39–2.24)	0.870	1.02 (0.42–2.51)	0.966
			dominant	0.80 (0.24–2.69)	0.715	0.89 (0.26–3.07)	0.849
			recessive	1.16 (0.23–5.83)	0.857	1.35 (0.26–7.09)	0.723
rs1880481	3/3/6 (0.38)	6/36/60 (0.24)	allelic	1.95 (0.80–4.74)	0.135	—	—
			additive	1.85 (0.78–4.36)	0.160	1.82 (0.76–4.31)	0.177
			dominant	1.43 (0.43–4.74)	0.560	1.44 (0.43–4.90)	0.555
			recessive	5.33 (1.14–25.01)	**0.034**	5.05 (1.02–25.03)	**0.048**
rs3864004	2/4/6 (0.33)	3/40/58 (0.23)	allelic	1.70 (0.68–4.21)	0.251	—	—
			additive	1.78 (0.68–4.68)	0.240	1.78 (0.64–4.94)	0.270
			dominant	1.35 (0.41–4.47)	0.625	1.21 (0.35–4.10)	0.765
			recessive	6.53 (0.97–43.85)	0.053	10.94 (1.24–96.68)	0.031
rs3755557	1/2/9 (0.17)	5/27/70 (0.18)	allelic	0.90 (0.29–2.80)	0.859	—	—
			additive	0.91 (0.32–2.65)	0.868	0.94 (0.32–2.79)	0.914
			dominant	0.73 (0.18–2.88)	0.652	0.75 (0.19–3.01)	0.681
			recessive	1.76 (0.19–16.5)	0.619	1.98 (0.20–20.00)	0.562

[a]In the order of homozygote / heterozygote / wild type.
[b]Adjusted for age.
Abbreviations: MAF, minor allele frequency; OR, odds ratio; CI, confidence interval; NA, not applicable. *P* value < 0.05 is shown in bold.

Supplementary Table S7: Association between SNPs in WNT/β-catenin pathway genes and acute grade 3–4 radiation-induced oral mucositis

SNP	Genotype distribution[a]		Model	Unadjusted		Adjusted[b]	
	Grade 3–4 (MAF)	Grade 0–2 (MAF)		OR (95% CI)	*P*	OR (95% CI)	*P*
rs10487362	21/45/23 (0.49)	22/46/31 (0.45)	allelic	1.15 (0.76–1.72)	0.507	—	—
			additive	1.14 (0.77–1.71)	0.513	1.17 (0.78–1.76)	0.443
			dominant	1.31 (0.69–2.47)	0.408	1.35 (0.71–2.56)	0.364
			recessive	1.08 (0.55–2.14)	0.823	1.12 (0.56–2.23)	0.743
rs2896218	6/45/38 (0.32)	4/42/53 (0.25)	allelic	1.39 (0.89–2.19)	0.146	—	—
			additive	1.47 (0.90–2.40)	0.120	1.5 (0.91–2.46)	0.110
			dominant	1.55 (0.87–2.75)	0.138	1.63 (0.91–2.93)	0.102
			recessive	1.72 (0.47–6.29)	0.415	1.50 (0.40–5.58)	0.542
rs6947329	23/38/27 (0.48)	24/47/28 (0.48)	allelic	0.99 (0.66–1.49)	0.961	—	—
			additive	0.99 (0.67–1.46)	0.963	1.01 (0.68–1.49)	0.979
			dominant	0.89 (0.47–1.67)	0.719	0.89 (0.47–1.68)	0.721
			recessive	1.11 (0.57–2.14)	0.766	1.16 (0.59–2.25)	0.674
rs454886	17/43/29 (0.43)	9/49/41 (0.34)	allelic	1.49 (0.98–2.26)	0.061	—	—
			additive	1.52 (0.99–2.34)	0.058	1.57 (1.01–2.43)	**0.045**
			dominant	1.46 (0.81–2.66)	0.212	1.50 (0.82–2.73)	0.191
			recessive	2.36 (0.99–5.61)	0.052	2.54 (1.06–6.13)	**0.038**
rs1880481	7/32/50 (0.26)	5/37/57 (0.24)	allelic	1.12 (0.70–1.79)	0.637	—	—
			additive	1.12 (0.70–1.78)	0.639	1.16 (0.72–1.85)	0.549
			dominant	1.06 (0.59–1.89)	0.847	1.1 (0.61–1.97)	0.752
			recessive	1.61 (0.49–5.25)	0.434	1.69 (0.51–5.61)	0.390
rs3864004	4/38/47 (0.26)	3/40/55 (0.23)	allelic	1.14 (0.71–1.82)	0.595	—	—
			additive	1.16 (0.70–1.92)	0.570	1.22 (0.73–2.04)	0.447
			dominant	1.14 (0.64–2.03)	0.650	1.2 (0.67–2.15)	0.546
			recessive	1.49 (0.32–6.85)	0.608	1.73 (0.37–8.14)	0.490
rs3755557	7/24/58 (0.21)	2/24/73 (0.14)	allelic	1.65 (0.96–2.82)	0.067	—	—
			additive	1.57 (0.94–2.63)	0.086	1.62 (0.96–2.73)	0.071
			dominant	1.50 (0.80–2.80)	0.203	1.57 (0.83–2.95)	0.166
			recessive	4.14 (0.84–20.48)	0.082	4.21 (0.84–21.04)	0.080

[a]In the order of homozygote / heterozygote / wild type.
[b]Adjusted for BMI.
Abbreviations: MAF, minor allele frequency; OR, odds ratio; CI, confidence interval. P value < 0.05 is shown in bold.

Supplementary Table S8: Association between SNPs in WNT/β-catenin pathway genes and acute grade 3–4 radiation-induced dermatitis

SNP	Genotype distribution[a]		Model	OR (95% CI)	*P*
	Grade 3–4 (MAF)	Grade 0–2 (MAF)			
rs10487362	3/6/7 (0.38)	40/85/47 (0.48)	allelic	0.65 (0.31–1.37)	0.257
			additive	0.66 (0.31–1.38)	0.267
			dominant	0.48 (0.17–1.37)	0.172
			recessive	0.46 (0.18–1.17)	0.102
rs2896218	0/7/9 (0.22)	10/80/82 (0.29)	allelic	0.68 (0.29–1.63)	0.388
			additive	0.65 (0.26–1.63)	0.356
			dominant	0.71 (0.25–1.99)	0.513
			recessive	2.30 (0.62–8.55)	0.214
rs6947329	4/6/5 (0.47)	43/79/50 (0.48)	allelic	0.95 (0.45–2.01)	0.891
			additive	0.95 (0.47–1.95)	0.896
			dominant	0.82 (0.27–2.52)	0.729
			recessive	0.88 (0.39–1.95)	0.746
rs454886	1/7/8 (0.28)	25/85/62 (0.39)	allelic	0.61 (0.27–1.35)	0.216
			additive	0.59 (0.26–1.35)	0.212
			dominant	0.56 (0.20–1.58)	0.274
			recessive	1.25 (0.49–3.19)	0.649
rs1880481	1/6/9 (0.25)	11/63/98 (0.25)	allelic	1.02 (0.44–2.35)	0.971
			additive	1.02 (0.44–2.33)	0.971
			dominant	1.03 (0.37–2.89)	0.955
			recessive	1.10 (0.28–4.25)	0.893
rs3864004	0/8/8 (0.25)	7/70/94 (0.25)	allelic	1.02 (0.44–2.37)	0.956
			additive	1.03 (0.42–2.52)	0.953
			dominant	1.22 (0.44–3.40)	0.703
			recessive	0.55 (0.06–4.68)	0.582
rs3755557	2/6/8 (0.31)	7/42/123 (0.31)	allelic	2.34 (1.05–5.21)	**0.033**
			additive	2.13 (1.00–4.55)	0.051
			dominant	2.51 (0.89–7.06)	0.081
			recessive	0.40 (0.05–3.25)	0.388

[a]In the order of homozygote / heterozygote / wild type.
Abbreviations: MAF, minor allele frequency; OR, odds ratio; CI, confidence interval. *P* value < 0.05 is shown in bold.

Supplementary Table S9: Association between SNPs in WNT/β-catenin pathway genes and acute grade 3–4 radiation-induced dermatitis myelosuppression

SNP	Genotype distribution[a]		Model	Unadjusted		Adjusted[b]	
	Grade 3–4 (MAF)	Grade 0–2 (MAF)		OR (95% CI)	*P*	OR (95% CI)	*P*
rs10487362	6/29/9 (0.47)	37/62/45 (0.47)	allelic	0.98 (0.60–1.57)	0.917	—	—
			additive	0.98 (0.61–1.56)	0.919	0.95 (0.58–1.56)	0.849
			dominant	1.77 (0.78–3.99)	0.170	1.79 (0.77–4.14)	0.175
			recessive	0.46 (0.18–1.17)	0.102	0.42 (0.16–1.11)	0.081
rs2896218	4/23/17 (0.35)	6/64/74 (0.26)	allelic	1.52 (0.91–2.53)	0.108	—	—
			additive	1.64 (0.93–2.87)	0.086	1.54 (0.86–2.75)	0.143
			dominant	1.68 (0.84–3.35)	0.141	1.63 (0.8–3.33)	0.182
			recessive	2.30 (0.62–8.55)	0.214	1.88 (0.48–7.34)	0.362
rs6947329	10/22/11 (0.49)	37/63/44 (0.48)	allelic	1.05 (0.65–1.70)	0.836	—	—
			additive	1.05 (0.66–1.66)	0.843	1.06 (0.66–1.70)	0.808
			dominant	1.28 (0.59–2.77)	0.531	1.45 (0.65–3.21)	0.363
			recessive	0.88 (0.39–1.95)	0.746	0.80 (0.35–1.82)	0.588
rs454886	7/18/19 (0.36)	19/74/51 (0.39)	allelic	0.90 (0.55–1.47)	0.670	—	—
			additive	0.89 (0.54–1.48)	0.664	0.81 (0.48–1.37)	0.441
			dominant	0.72 (0.36–1.44)	0.352	0.64 (0.31–1.32)	0.230
			recessive	1.25 (0.49–3.19)	0.649	1.08 (0.41–2.86)	0.881
rs1880481	3/13/28 (0.22)	9/56/79 (0.26)	allelic	0.80 (0.45–1.41)	0.435	—	—
			additive	0.80 (0.45–1.41)	0.439	0.65 (0.36–1.17)	0.152
			dominant	0.69 (0.35–1.39)	0.305	0.54 (0.26–1.13)	0.103
			recessive	1.10 (0.28–4.25)	0.893	0.78 (0.19–3.10)	0.720
rs3864004	1/15/27 (0.20)	6/63/75 (0.26)	allelic	0.70 (0.39–1.27)	0.236	—	—
			additive	0.67 (0.35–1.25)	0.207	0.53 (0.27–1.03)	0.059
			dominant	0.64 (0.32–1.30)	0.218	0.5 (0.24–1.05)	0.067
			recessive	0.55 (0.06–4.68)	0.582	0.38 (0.04–3.27)	0.376
rs3755557	1/12/31 (0.16)	8/36/100 (0.18)	allelic	0.86 (0.45–1.64)	0.643	—	—
			additive	0.87 (0.47–1.61)	0.662	0.83 (0.44–1.56)	0.560
			dominant	0.95 (0.46–1.99)	0.899	0.90 (0.42–1.95)	0.793
			recessive	0.40 (0.05–3.25)	0.388	0.36 (0.04–3.05)	0.350

[a]In the order of / heterozygote / wild type.

[b]Adjusted for T stage and chemotherapy.

Abbreviations: MAF, minor alle homozygote le frequency; OR, odds ratio; CI, confidence interval. *P* value < 0.05 is shown in bold.

Supplementary Table S10: Primer sequences for 9 SNPs in genotyping

SNP-ID	Forward primer	Reverse primer	Extension primer
**rs10487362**	ACGTTGGATGTGCCCCTCCAAGAGTAAAAG	ACGTTGGATGGTGAACTAATGCATCAGCAG	ATTCATCAGCAGACTATATCTATCG
**rs2896218**	ACGTTGGATGCTAGGAATGTCTTCCTTTTG	ACGTTGGATGAATTCCAACACAGAGCGGAC	ACAGTCACGAGGCAT
**rs6947329**	ACGTTGGATGATCATTTCCAGCCTCTCCCC	ACGTTGGATGACCACAAGTACCTTCAAGGC	CCTCCCACCCTAGGTTCTAATTT
**rs3777860**	ACGTTGGATGACCAGGAAAGTGGTGTATGC	ACGTTGGATGACTGCAAGCACTCATGCTGG	GGGGAAGGTCACTGGCACATTTCAA
**rs454886**	ACGTTGGATGTATGCAGTAAGAGAAGAAG	ACGTTGGATGGTCAGCTCTCCTTTCTTTGG	CTAGGCCTCATTTTGTATCATATAAAAC
**rs4135385**	ACGTTGGATGGGAGTTACTTGTTCCTTTTG	ACGTTGGATGAGCAAGGAAGAATAGAAAGC	CATGAAAATTCTCAAAAGTACACTAA
**rs1880481**	ACGTTGGATGCCCGTGCTTAATTCTTAGTC	ACGTTGGATGGCTGTCATTTCTCCCATAAC	CTCCCATAACACTCATTCTAATTTA
**rs3864004**	ACGTTGGATGAGGACTTGTTGAATTGCGGG	ACGTTGGATGTTCTGTCCCCACTCACGAAG	GTGAACTCTCCGTAGAA
**rs3755557**	ACGTTGGATGCCTGCAGAGTCATCTCTTTC	ACGTTGGATGGTTTCAAAGCAAGAGCCAGG	CTACTCTGATCAAATATAGGTCCTTT
